# Reactivity of Metal-Free and Metal-Associated Amyloid-*β* with Glycosylated Polyphenols and Their Esterified Derivatives

**DOI:** 10.1038/srep17842

**Published:** 2015-12-10

**Authors:** Kyle J. Korshavn, Milim Jang, Yeon Ju Kwak, Akiko Kochi, Silvia Vertuani, Anirban Bhunia, Stefano Manfredini, Ayyalusamy Ramamoorthy, Mi Hee Lim

**Affiliations:** 1 Department of Chemistry, University of Michigan, Ann Arbor, Michigan 48109-1055, United States; 2 Department of Chemistry, Ulsan National Institute of Science and Technology (UNIST), Ulsan 44919, Korea; 3 Department of Life Sciences and Biotechnology, University of Ferrara, I-44121 Ferrara, Italy; 4 Biophysics, University of Michigan, Ann Arbor, Michigan 48109-1055, United States; 5 Department of Biophysics, Bose Institute, P-1/12 CIT Scheme VII(M), Kolkata 700054, India

## Abstract

Both amyloid-*β* (A*β*) and transition metal ions are shown to be involved in the pathogenesis of Alzheimer’s disease (AD), though the importance of their interactions remains unclear. Multifunctional molecules, which can target metal-free and metal-bound A*β* and modulate their reactivity (*e.g*., A*β* aggregation), have been developed as chemical tools to investigate their function in AD pathology; however, these compounds generally lack specificity or have undesirable chemical and biological properties, reducing their functionality. We have evaluated whether multiple polyphenolic glycosides and their esterified derivatives can serve as specific, multifunctional probes to better understand AD. The ability of these compounds to interact with metal ions and metal-free/-associated A*β*, and further control both metal-free and metal-induced A*β* aggregation was investigated through gel electrophoresis with Western blotting, transmission electron microscopy, UV-Vis spectroscopy, fluorescence spectroscopy, and NMR spectroscopy. We also examined the cytotoxicity of the compounds and their ability to mitigate the toxicity induced by both metal-free and metal-bound A*β*. Of the polyphenols investigated, the natural product (**Verbascoside**) and its esterified derivative (**VPP**) regulate the aggregation and cytotoxicity of metal-free and/or metal-associated A*β* to different extents. Our studies indicate **Verbascoside** represents a promising structure for further multifunctional tool development against both metal-free A*β* and metal-A*β*.

Alzheimer’s disease (AD) is a growing concern for global public health, spurred on, in part, by a lack of effective treatments or cures^1^. While potential therapeutics have been developed for AD, their clinical success has been hindered by a limited molecular level understanding of the disease’s etiology^2^,^3^,^4^. AD is commonly considered a protein misfolding disease characterized by the presence of protein aggregates, including senile plaques composed primarily of amyloid-*β* (two main A*β* forms, A*β*_40_ and A*β*_42_)^4^,^5^,^6^. A*β* undergoes a progressive aggregation process, advancing from a small, intrinsically disordered peptide to intermediate oligomers of various sizes and structures, finally forming extended fibers; individual aggregates are believed to have varying degrees of toxicity and relevance in AD^4^,^6^,^7^.

The senile plaques in the AD-affected brain have also been shown to contain elevated concentrations of transition metals, specifically Cu, Zn, and Fe, which suggests that these metal ions interact with and alter the aggregation of A*β *4,8,9,10. Understanding the interactions between metal ions and A*β in vitro* could help elucidate potentially toxic mechanisms of both factors in AD^10^,^11^,^12^. These metal ions could accelerate the aggregation of A*β* while simultaneously generating a variety of aggregates which may have biological functions distinct from those formed in the absence of metals^9^,^10^. Additionally, the binding of A*β* to redox active metals (*i.e.*, Cu) can facilitate redox cycling and lead to the production of reactive oxygen species (ROS) resulting in an oxidative stress environment, a known characteristic of the AD-afflicted brain^12^,^13^,^14^. The effects of these interactions *in vivo* remain unclear, however; in-depth understanding is hindered by the multifactorial nature of the disease which makes it difficult to identify and quantify the influence of any of the potentially causative agents.

The application of chemical probes which can modulate the various factors associated with AD (*e.g.*, A*β,* metal ions) may advance our understanding of the disease and uncover different toxic factors by isolating individual potential culprits. Unfortunately, there remains a lack of understanding about the relationship between small molecules and their subsequent biological functions with regards to the multiple aspects associated with AD. It is believed that hydrophobic interactions drive early stages of A*β* aggregation and that compounds with similarly hydrophobic regions may effectively disrupt these aggregation-promoting forces and act as modulators of amyloid formation; some compounds of this nature have been investigated previously^6^. It is unclear, though, which chemical moieties on these compounds may be the most potent at altering this process. To increase the specificity of anti-amyloidogenic compounds, multifunctional compounds have been designed to target and modulate additional aspects associated with AD (*i.e.*, metal-associated A*β* (metal–A*β*), ROS) along with metal-free Aβ simultaneously^15^,^16^,^17^. Compounds known to interact with A*β* have been appended with known metal binding moieties to generate molecules capable of targeting both metal-free A*β* and metal–A*β*, and these multifunctional compounds have demonstrated an ability to modulate both factors to differing extents^15^,^16^. As was the case with A*β* interaction, however, rational design of these metal binding moieties is hindered by a limited understanding of how these chelating agents function in the complex AD environment. Additionally, while it is desirable for compounds to also mediate oxidative stress, both through the modulation of ROS and free radicals, this function is similarly difficult to rationally incorporate into a structural entity. Efforts have previously been made to design multifunctional compounds for investigating the role of metal-free A*β*, metal-A*β,* and ROS in AD^18^, but the advancements in the field are generally slowed by limited information on the structure-function relationships between small molecules and their ability to regulate these disease-related features.

In order to gain better insight to this complex disease and to broaden current understanding of the connection between chemical structure and its function in the presence of factors associated with AD, three naturally occurring polyphenolic glycosides (**Phlorizin**, **Verbascoside**, and **Rutin**; Fig. 1) were chosen for a selective reactivity study towards both metal-free A*β* and metal-A*β*. The investigation of naturally occurring compounds gives the substantial advantage of a minimal toxicity profile and a significant amount of background information from traditional medicine. Natural polyphenolic products have been previously shown to possess potential anti-amyloidogenic activity^19^,^20^. Both **Verbascoside** and **Rutin** have demonstrated the capacity to alter the aggregation and toxicity of metal-free A*β* aggregation towards nontoxic species^21^,^22^,^23^,^24^, and phloretin, the non-glycosidic version of **Phlorizin**, has exhibited an ability to prevent membrane-associated aggregation of A*β*^25^. While these preliminary studies examined aggregation in the absence of metal ions, the presence of known metal interaction moieties, phenol and catechol groups in **Phlorizin** and **Verbascoside**/**Rutin**, respectively, suggests that these compounds may be capable of simultaneously interacting with both A*β* and metal ions^26^,^27^,^28^,^29^. Furthermore, all three compounds are known antioxidants, indicating that they could also help mitigate oxidative stress associated with the AD-affected brain^30^,^31^,^32^. Finally, these compounds are naturally glycosylated, which has been proposed to improve bioavailability and distribution in the brain^33^,^34^,^35^, as well as redirect the folding of metal-free A*β* species^36^,^37^. Combined, these structural and chemical features suggest that **Phlorizin**, **Verbascoside**, and **Rutin** possess unique chemical features desirable for a probe to target multiple factors (*i.e.*, metal-free and metal-bound A*β*) and mitigate their toxicity leading to AD.

To provide insight into structure-activity relationships, selectively esterified derivatives, **F2**, **VPP**, and **R2** (Fig. 1), were prepared and investigated alongside their parent compounds^30^,^31^,^32^. Esterification could provide multiple benefits in the quest for an effective multifunctional probe while also acting as a general proof of concept that a prodrug approach is a viable method in the search for molecular tools in AD research. Ester formation may improve trafficking across lipid bilayers and facilitate targeted delivery of unprotected compounds within cells following cleavage by esterases; for these reasons, it is a commonly used modification in prodrug design^38^. The ester groups may also improve the ability of the compounds to passively diffuse across the blood brain barrier (BBB) due to greater lipophilicity^16^,^33^. Finally, the increased hydrophobicity could promote interactions between the compounds and the more aggregation prone regions of the A*β* sequence, which are similarly hydrophobic^4^. Overall, we believe that esterification may tune the ability of these three compounds to interact with both metal-free A*β* and metal–A*β* species while simultaneously improving the bioavailability, making them more suitable for future *in vivo* applications than their non-esterified counterparts. Using these six compounds, we aim to further expand the understanding of structure-function relationships between multifunctional probes towards both metal-free A*β* and metal-A*β* through a detailed, molecular level characterization.

## Results

### Influence of Polyphenols on Metal-Free and Metal-Induced Aggregation *In Vitro*

**Figure 1:**
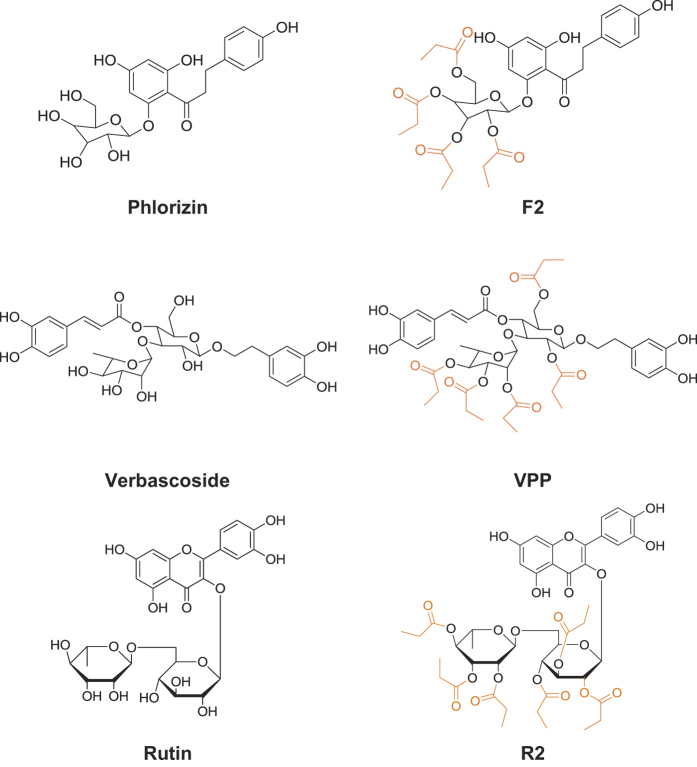
Chemical structures of glycosylated polyphenols and their derivatives. **Phlorizin**, 1-(2,4-dihydroxy-6-(((2*S*,3*R*,4*S*,5*S*,6*R*)-3,4,5-trihydroxy-6-(hydroxymethyl)tetrahydro-2*H*-pyran-2-yl)oxy)phenyl)-3-(4-hydroxyphenyl)propan-1-one; **Verbascoside**, (2*R*,3*R*,4*R*,5*R*,6*R*)-6-(3,4-dihydroxyphenethoxy)-5-hydroxy-2-(hydroxymethyl)-4-(((2*S*,3*R*,4*R*,5*R*)-3,4,5-trihydroxy-6-methyltetrahydro-2*H*-pyran-2-yl)oxy)tetrahydro-2*H*-pyran-3-yl (*E*)-3-(3,4-dihydroxy phenyl)acrylate; **Rutin**, 2-(3,4-dihydroxyphenyl)-5,7-dihydroxy-3-(((2*S*,3*R*,4*S*,5*S*,6*R*)-3,4,5-trihydroxy-6-((((2*R*,3*R*,4*R*,5*R*,6*S*)-3,4,5-trihydroxytetrahydro-2*H*-pyran-2-yl)oxy)methyl)tetra hydro-2*H*-pyran-2-yl)oxy)-4*H*-chromen-4-one; **F2**, (2*S*,3*R*,4*S*,5*R*,6*R*)-2-(3,5-dihydroxy-2-(3-(4-hydroxyphenyl)propanoyl)phenoxy)-6-((propionyloxy)methyl)tetrahydro-2*H*-pyran-3,4,5-triyl tripropionate; **VPP**, (2*S*,3*R*,4*R*,5*S*,6*S*)-2-(((2*R*,3*R*,4*S*,5*R*,6*R*)-2-(3,4-dihydroxyphenethoxy)-5-(((*E*)-3-(3,4-dihydroxyphenyl)acryloyl)oxy)-3-(propionyloxy)-6-((propionyloxy)methyl)tetrahydro-2*H*-pyran-4-yl)oxy)-6-methyltetrahydro-2*H*-pyran-3,4,5-triyl tripropionate; **R2**, (2*R*,3*R*,4*R*,5*S*,6*S*)-2-(((2*R*,3*R*,4*S*,5*R*,6*S*)-6-((2-(3,4-dihydroxyphenyl)-5,7-dihydroxy-4-oxo-4*H*-chromen-3-yl)oxy)-3,4,5-tris(propionyloxy)tetrahydro-2*H*-pyran-2-yl)methoxy)-6-methyl tetrahydro-2*H*-pyran-3,4,5-triyl tripropionate.

**Figure 2:**
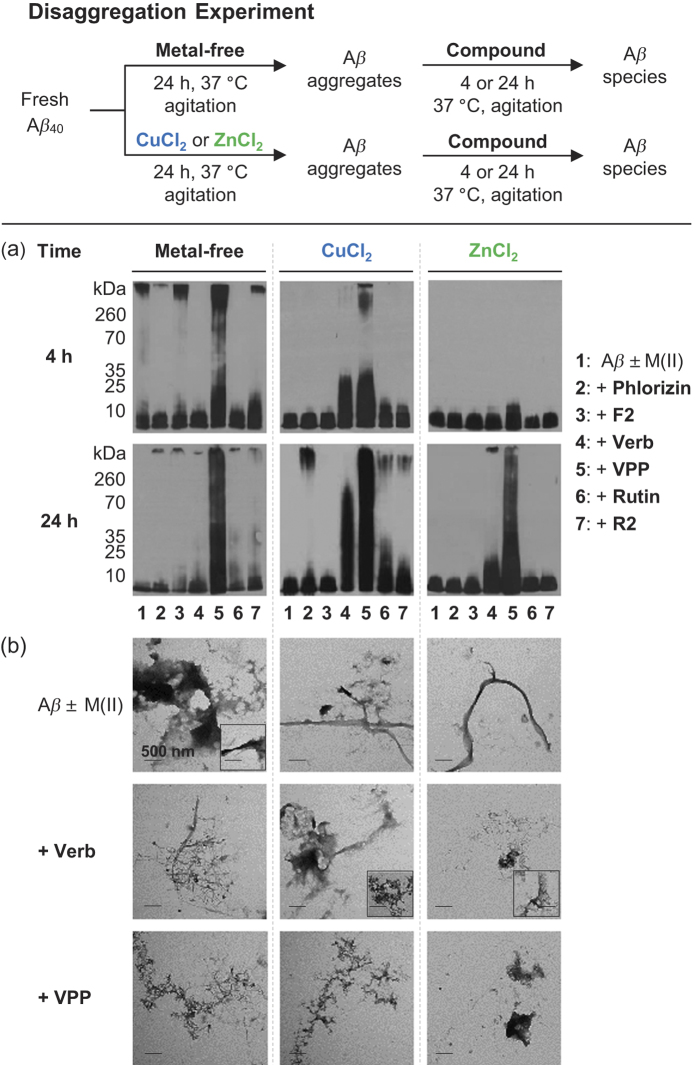
Modulation of preformed, metal-free and metal-induced A*β*_40_ aggregates by Phlorizin, F2, Verbascoside, VPP, Rutin, or R2. A scheme of sample preparation for disaggregation experiments with A*β*_40_ (top). (**a**) Analysis of the resultant A*β*_40_ species by gel electrophoresis using Western blotting with an anti-A*β* antibody (6E10). (**b**) TEM images of the morphologies of the resultant A*β* species from the samples that were incubated for 24 h. Experimental conditions: [A*β*_40_] = 25 μM; [CuCl_2_ or ZnCl_2_] = 25 μM; [compound] = 50 μM; 4 or 24 h incubation; pH 6.6 (for Cu(II) samples) or pH 7.4 (for metal-free and Zn(II) samples); 37 °C; constant agitation.

**Figure 3:**
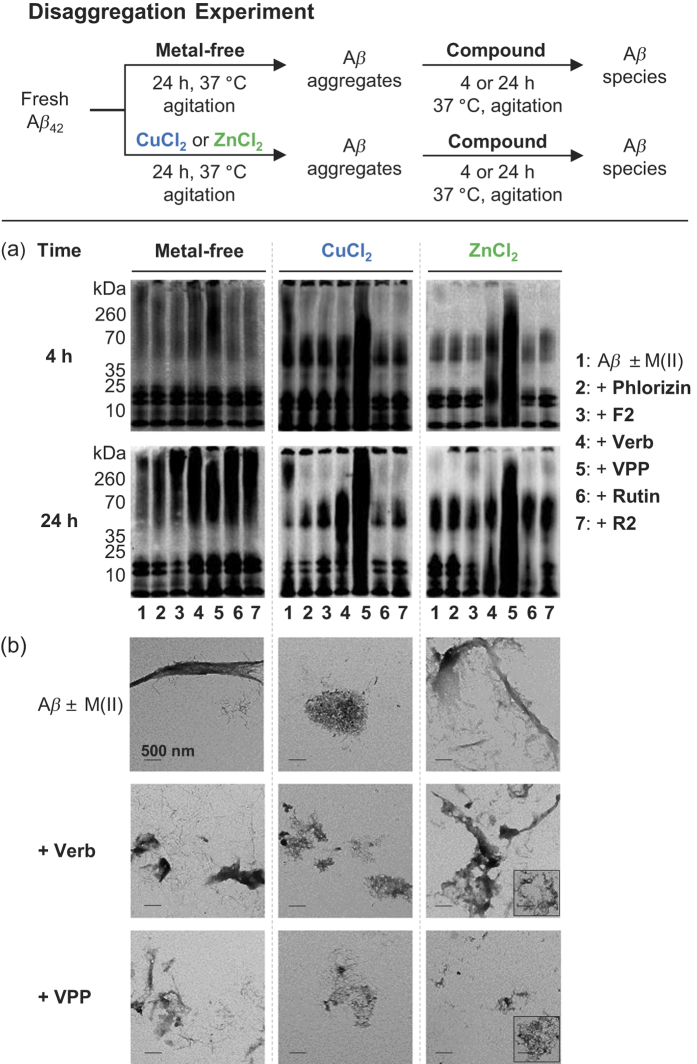
Influence of Phlorizin, F2, Verbascoside, VPP, Rutin, or R2 on pre-generated metal-free and metal-induced A*β*_42_ aggregates. A scheme of sample preparation for disaggregation experiments with A*β*_42_ (top). (**a**) Analysis of the resultant A*β*_42_ species by gel electrophoresis using Western blot with an anti-A*β* antibody (6E10). (**b**) TEM images of the morphologies of the resultant A*β* species from the samples that were incubated for 24 h. Experimental conditions: [A*β*_42_] = 25 μM; [CuCl_2_ or ZnCl_2_] = 25 μM; [compound] = 50 μM; 4 or 24 h incubation; pH 6.6 (for Cu(II) samples) or pH 7.4 (for metal-free and Zn(II) samples); 37 °C; constant agitation.

Initially, to investigate the effects of the six polyphenols (Fig. 1) on the structure and formation of both metal-free A*β* and metal–A*β* aggregation pathways, disaggregation (Figs 2 and 3, and S1) and inhibition (Fig. S2) experiments were performed. Both the more prevalent A*β*_40_ and the more aggregation-prone A*β*_42_ isoforms were employed in both inhibition and disaggregation studies^4^. For disaggregation experiments, A*β* was allowed to aggregate in either the absence or presence of either CuCl_2_ or ZnCl_2_ for 24 h to form aggregated species. The compounds were then added to these aggregates and incubated for either 4 or 24 h to determine their ability to redirect the size and structure of pre-aggregated A*β* species. In inhibition experiments, A*β* (for metal-A*β* samples, A*β* samples were treated with CuCl_2_ or ZnCl_2_) and the compounds were incubated for either 4 or 24 h to identify how they are capable of modulating the early steps in aggregation of both metal-free A*β* and metal-A*β*. Gel electrophoresis with Western blot (gel/Western blot) using an anti-A*β* antibody (6E10) and TEM were utilized to visualize the size distribution and morphology, respectively, of the resultant A*β* species upon treatment with polyphenols in the absence and presence of metal ions for both disaggregation and inhibition experiments^18^,^39^.

Neither **Phlorizin** nor **F2** demonstrated a significant ability to interact with metal-free A*β* or metal–A*β* species in either a disaggregatory or inhibitory manner. In disaggregation experiments, gel/Western blot revealed that neither of these compounds was able to transform preformed A*β* aggregates regardless of A*β* isoforms or metal presence (Figs 2a and 3a, lanes 2 and 3). **Phlorizin** did appear to slightly change the morphology of species to marginally more disordered than untreated fibrils; however, large and extended A*β* aggregates were observed with and without metal ions (Fig. S1). A similar lack of reactivity was observed in inhibition experiments (Fig. S2, lanes 2 and 3). These two compounds appear unable to influence A*β*_40_ and A*β*_42_ aggregation behaviors regardless of the presence or absence of metal ions.

Both **Rutin** and **R2** showed mild reactivity with metal-free A*β* and/or metal–A*β*. Under disaggregation conditions, **R2** produced low MW aggregates of A*β*_40_ (<15 kDa) after 4 h in the absence of metal ions; after 24 h in the absence of metal ions, both **Rutin** and **R2** triggered relatively low MW aggregates (<35 kDa) (Fig. 2a, lanes 6 and 7). The morphology of these species was slightly more compact that that of untreated A*β*_40_ aggregates (Fig. S1). A relatively similar range of A*β*_40_ aggregates was generated by both **Rutin** and **R2** when CuCl_2_ was present, after 24 h (Fig. 2a), and their morphology was even more compact than in the absence of metal ions (Fig. S1). Neither **Rutin** nor **R2** appeared to have any impact on preformed Zn(II)–A*β*_40_ aggregates. The two compounds displayed a minimal or no effect upon preformed A*β*_42_ aggregates, regardless of the presence or absence of metal ions (Fig. 3a, lanes 6 and 7). **Rutin** and **R2** presented a modest ability to redirect Cu(II)-induced aggregation of A*β*_40_ or A*β*_42_ after 24 h incubation in inhibition studies (Fig. S2, lanes 6 and 7). This suggests that both compounds have mild reactivity towards preformed A*β* aggregates, favoring those formed in the presence of CuCl_2_.

Unlike the other four compounds, both **Verbascoside** and **VPP** were able to control A*β*_40_ and A*β*_42_ aggregation, though to different extents. When preformed, metal-free A*β*_40_ aggregates were treated with **VPP** for 4 h, a wide MW range (10–260 kDa) of species was formed (Fig. 2a, lane 5). These species were still observed after 24 h incubation. **Verbascoside**, the non-esterified parent compound of **VPP**, did not indicate any reactivity against preformed metal-free A*β*_40_ aggregates. Both compounds did demonstrate an ability to disaggregate Cu(II)–A*β*_40_ aggregates, however (Fig. 2, lanes 4 and 5). At the early incubation time point (*i.e.*, 4 h) the two compounds mainly generated relatively low MW (<35 kDa) aggregates, evident by gel/Western blot. At the later incubation time point (*i.e.*, 24 h), both compounds were capable of producing much wider MW ranges of Cu(II)–A*β*_40_ species (Fig. 2a, lanes 4 and 5). In the case of Zn(II)-containing aggregates; the initial disaggregation by **Verbascosde** and **VPP** was slower still. The A*β*_40_ sample treated with **Verbascoside** for 24 h exhibited smaller-sized species while **VPP** triggered a wider range of aggregates, similar to that observed in both the absence of metals and the presence of Cu(II) (Fig. 2a). For all aggregates, both **Verbascoside** and **VPP** were able to convert large extended fibrils into smaller, generally amorphous structures (Fig. 2b). This same reactivity trend was observed when **Verbascoside** and **VPP** were added to metal-free and metal-associated, preformed A*β*_42_ aggregates (Fig. 3). These compounds were capable of slightly altering A*β*_42_ aggregates in the absence of metal ions. Additionally, **VPP** generated various-sized aggregates of Zn(II)–A*β*_42_ after 4 h only, a process which required 24 h with A*β*_40_ (Fig. 3a). Similar to A*β*_40_, unstructured A*β*_42_ aggregates induced by treatment with the two compounds were shown by TEM (Fig. 3b).

**Verbascoside** and **VPP** also displayed reactivity towards A*β*_40_ and A*β*_42_ in inhibition experiments (Fig. S2, lanes 4 and 5). Only **VPP** affected the aggregation of metal-free A*β*_40_/A*β*_42_, which was evident just after 4 h with both isoforms. Both **Verbascoside** and **VPP** demonstrated inhibitory activity towards metal-A*β*_40_/A*β*_42_. In the presence of Cu(II), the two compounds promoted a variety of aggregates to different extents after 4 h incubation; **Verbascoside** generated a smaller MW range of aggregates than **VPP**, suggesting that their activity may be size and/or conformation dependent. A similar trend in A*β* aggregate size was observed in the presence of Zn(II) when **Verbascoside** and **VPP** were introduced (Fig. S2). **Verbascoside** and **VPP** presented the similar time dependence in the presence of Zn(II) for the inhibition of A*β*_40_ aggregation as they did with A*β*_40_ disaggregation; their reactivity towards Zn(II)–A*β*_40_ was noticeable only after 24 h (Fig. S2a). This suggests that the timescale of reactivity may be dependent upon solution conditions, specifically the metal(s) present in solution and the morphology of aggregates, at a given time. Taken together, the results from these disaggregation and inhibition experiments suggest that **Verbascoside** and **VPP** have a distinct capacity to modulate the aggregation of metal–A*β* (as well as metal-free A*β* mainly in the case of **VPP**) which is generally absent for the other four compounds analyzed in this study. It is interesting to observe that among the selected polyphenols the reactive **Verbascoside** and **VPP** both feature distinctive double catechol moieties, bearing the two characteristic *ortho*-hydroxy groups. Meanwhile, **Rutin** and **R2** which only show mild reactivity have a single catechol moiety. **Phloririzin** and **F2** have no catechol moieties while also presenting no noticeable reactivity with A*β* species. This points to a potential role for the catechol moiety in the activity of these compounds. Additionally, from this set of compounds, it appears that structural modification of the sugar moiety (*i.e.*, selective esterification) can drastically alter the function of the parent compound, as was seen for both **Verbascoside**/**VPP** and **Rutin**/**R2**.

### Metal Binding Properties of Polyphenols

**Figure 4:**
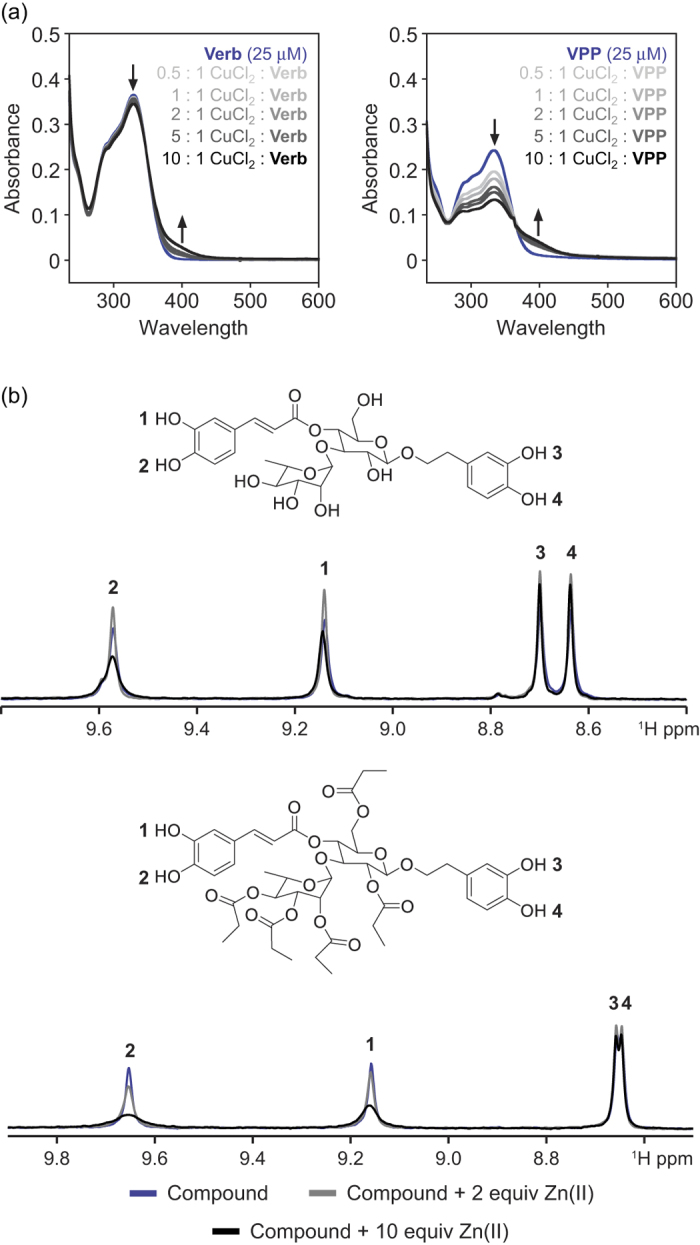
Metal binding studies of Verbascoside and VPP. (**a**) Cu(II) binding studies of **Verbascoside** (**Verb**) and **VPP** by UV-Vis. Samples were incubated for 2 h with or without Cu(II) (0.5–10 equiv) at pH 7.4 at room temperature. (**b**) Zn(II) binding studies of **Verbascoside** (top) and **VPP** (bottom) by ^1^H NMR. The samples of compounds (2 mM) were titrated with ZnCl_2_ (0, 2, and 10 equiv) in DMSO-*d*_*6*_ at room temperature.

In order to elucidate the molecular level interactions responsible for the varied anti-amyloidogenic activity of the polyphenols towards metal–A*β* species (*vide supra*), it is necessary to understand the potential binding of each compound with the component parts of system: metal ions and A*β* monomers/aggregates. The potential interaction between the polyphenols and metal ions was investigated first. The OH groups found on the aromatic rings of all six compounds could potentially interact with transition metals; the catechol moieties of **Verbascoside**, **VPP**, **Rutin**, and **R2** are especially likely to bind metal ions in a manner similar to previously reported polyphenols^20^,^28^,^40^,^41^,^42^. It has already been suggested that both **Verbascoside** and **Rutin** could interact with Cu(II) in solution^43^,^44^,^45^. The Cu(II) binding properties of **Phlorizin**/**F2**, **Verbascoside**/**VPP**, and **Rutin**/**R2** were examined by UV-Vis while the Zn(II) binding of **Verbascoside** and **VPP** was monitored by ^1^H NMR due to a lack of significant optical changes in the spectra when the ligand was treated with Zn(II) (Fig. 4, S3 and S4).

The UV-Vis spectra of the six polyphenols showed different levels of spectral changes following the titration of CuCl_2_ in buffered aqueous solution (pH 7.4; Fig. 4a and S3). Addition of CuCl_2_ to both **Verbascoside** and **VPP** in solution induced a slight change in the absorption bands at *ca*. 330 nm and 405 nm, implying potential interaction between the catechol moieties of both ligands and Cu(II) in solution (Fig. 4a)^46^. The spectral change is more drastic in **VPP** than in **Verbascoside**, which suggests that esterification of the OH groups in the sugar ring could reduce their competitive interaction with Cu(II) and promote binding by the catechol moieties. Neither compound presented significantly noticeable spectral shifts indicative of complex formation, however, suggesting that interaction of either **Verbascoside** or **VPP** with Cu(II) in solution could be weak. Previous studies on complex formation between **Verbascoside** and Cu(II) are unclear about the extent of complex formation, which indicates that the complex formation could depend upon experimental conditions (*i.e.*, buffer, salt concentration, pH)^43^,^47^. Addition of CuCl_2_ to solutions of either **Phlorizin** or **F2** induced no significant shift in spectral features, possibly due to the minimal interaction of the phenol moiety in the compounds for Cu(II) in solution (Fig. S3). The presence of Cu(II) did induce modest changes in the spectrum of **Rutin**, including a decrease in the primary peak at *ca*. 330 nm and an increase in a shoulder at *ca.* 425 nm (Fig. S3). This may be caused by the interaction between the catechol moiety and Cu(II). These shifts are similar, though of smaller magnitude, to those previously observed for **Rutin**-Cu(II) complexes^44^,^45^. Finally, **R2** exhibited slight spectral changes over the course of the titration, suggesting minimal or no interaction with Cu(II) in solution (Fig. S3), suggesting that esterification of **Rutin** could change the interaction between the ligand and Cu(II). These results suggest that only **Verbascoside**, **VPP**, and **Rutin** have an observable ability to interact with Cu(II) under the conditions of this study.

The interaction of **Verbascoside** and **VPP** with Zn(II) was also investigated by ^1^H NMR in DMSO-*d*_6_ (Fig. 4b). When Zn(II) was titrated into **Verbascoside** up to 2 equiv, all phenolic protons exhibited minor sharpening while titration up to 10 equiv resulted in selective broadening of the resonances associated with the caffeic acid moiety. The hydroxytyrosine phenolic protons showed minimal change upon addition of more Zn(II). Selective broadening suggests that Zn(II) preferentially associates with the catechol moiety in caffeic acid which causes proton exchange with residual water present in the solvent. A similar pattern was observed when Zn(II) was added to **VPP**. During the titration of **VPP**, the protons of the catechol group in caffeic acid broadened, suggesting interaction with the metal and subsequent proton exchange with the residual aqueous solvent. No other proton resonances demonstrate any shifts or broadening over the course of the titration for either compound (Fig. S4). This would suggest that in both **Verbascoside** and **VPP**, Zn(II) is observed to relatively weakly interact with the phenolic protons of the caffeic acid moiety over all other portions of the compounds, though weakly. Overall, both **Verbascoside** and **VPP** are shown to interact with both Cu(II) and Zn(II) with a modest affinity under the conditions employed in this study, implying that direct interaction with the metal ions could be partially responsible for the ability of these compounds to redirect metal–A*β* aggregation (*vide supra*). This is likely not the direct result of metal chelation, however, given the relatively strong affinity of A*β* for both metal ions^10^.

### Interaction of Polyphenols with Multiple A*β* Forms

**Figure 5:**
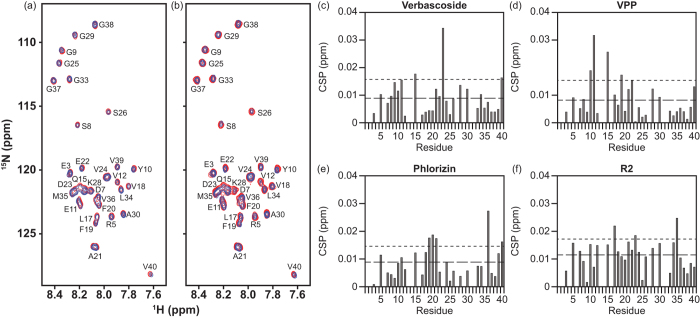
Ligand interaction with monomeric A*β*_40_ by 2D ^1^H/^15^N SOFAST-HMQC NMR (600 MHz). **Verbascoside** (**a**) and **VPP** (**b**) were titrated into a solution of uniformly ^15^N-labeled A*β*_40_ (80 μM in 20 mM PO_4_, 50 mM NaCl, pH 7.4, 7% D_2_O (v/v), 10 °C). Blue color represents the spectrum obtained upon addition of 10 equiv of the ligand to A*β*_40_ (red color: the spectrum of A*β*_40_ only). All spectra were aligned at G29 due to non-uniform broadening of the water peak as a reference. Chemical shift perturbation (CSP) between the 0 and 10 equiv of all resolved residues was calculated and plotted as a function of the amino acid residue number in A*β*_40_ for (**c**) **Verbascoside**, (**d**) **VPP**, (**e**) **Phlorizin**, and (**f**) **R2**. All CSP values are compared to the average (dashed line) and average + standard deviation (dotted line) for each titration. Values which exceed the average + standard deviation line are considered to be significant, suggesting potential interaction.

Given the ability of both **Verbascoside** and **VPP** to redirect the morphology and aggregation of A*β*, the direct interaction of the two, along with both **Phlorizin** and **R2**, with monomeric A*β*_40_ was investigated (Fig. 5 and S5). 2D band-Selective Optimized-Flip-Angle Short-Transient Heteronuclear Multiple Quantum Coherence (SOFAST-HMQC) NMR experiments were performed to identify potential residue-specific interactions between monomeric A*β* peptide and the compounds^48^. The chemical shift perturbation (CSP) for each resolvable residue was calculated by comparing the spectrum of ligand-free A*β*_40_ with that of A*β*_40_ in the presence of excess ligand (10 equiv) and these CSP values were compared to the average CSP values (Figs 5a, 5b and S4) to identify potentially favored interactions^20^,^28^. All four compounds induced some mild to moderate (0.02–0.035 ppm) chemical shifts in varying regions of the peptide. **Verbascoside** (Fig. 5c) triggered a relatively distinct shift in D23 and also weakly perturbed Q15 while **VPP** (Fig. 5d) caused some shifts in Y10, E11, Q15, or F20. The interactions of **Verbascoside** seem to favor both polar and charged residues that are centrally located in the sequence, suggesting that both dipole-dipole interactions and hydrogen bonding could direct potential ligand binding. **VPP**, however, perturbed a mixture of hydrophobic and hydrophilic residues, suggesting that the added acyl groups could encourage some nonpolar contacts during ligand interaction with A*β*. π–π stacking between **VPP** and the aromatic side chains of Y10 and/or F20 could also be responsible for the observed shifts. Despite causing shifts in unique residue resonances, both compounds alter residues near the purported metal binding site of A*β* (residues 1–16) suggesting that the compounds preferentially interact with the N-terminus and may be oriented in a manner which promotes interaction with metal ions when present in solution or could alter the conformation of the N-terminus of the peptide, disrupting the peptide’s ability to bind metal ions^4^,^8^,^9^,^10^. **Phlorizin** (Fig. 5e) caused minor chemical shifts in both the hydrophobic core (F19-A21) and the C-terminus (V36); nonpolar forces may be responsible for any prospective **Phlorizin**–A*β*_40_ interaction. **Rutin** (Fig. 5f) prompted small shifts in regions similar to those altered by **Phlorizin** (L17 and M35). These small and non-localized chemical shifts could be indicative of weak interaction and/or non-specific binding to the peptide by the two compounds. Both **Phlorizin** and **R2**, which show limited or no influence on amyloidogenesis, display potentially weak interactions with more C-terminal residues while the interaction of both **Verbascoside** and **VPP** with A*β* appear to be more centrally or N-terminally located. Due to the modest CSP values observed, these data may suggest a general preference for interaction rather than a conventional, structured binding site for all compounds examined. Our NMR studies, therefore, suggest a possible molecular mechanism responsible for their differing activities towards A*β* species *in vitro* (*vide supra*). Ligand association with the N-terminus (close to the metal binding site in A*β*) may be favorable in the development of multifunctional compounds to target metal-free and/or metal-associated A*β* species while interacting with the central hydrophobic residues of A*β* may also allow for general inhibition, as has been previously predicted^4^,^6^,^9^,^10^,^15^.

**Figure 6:**
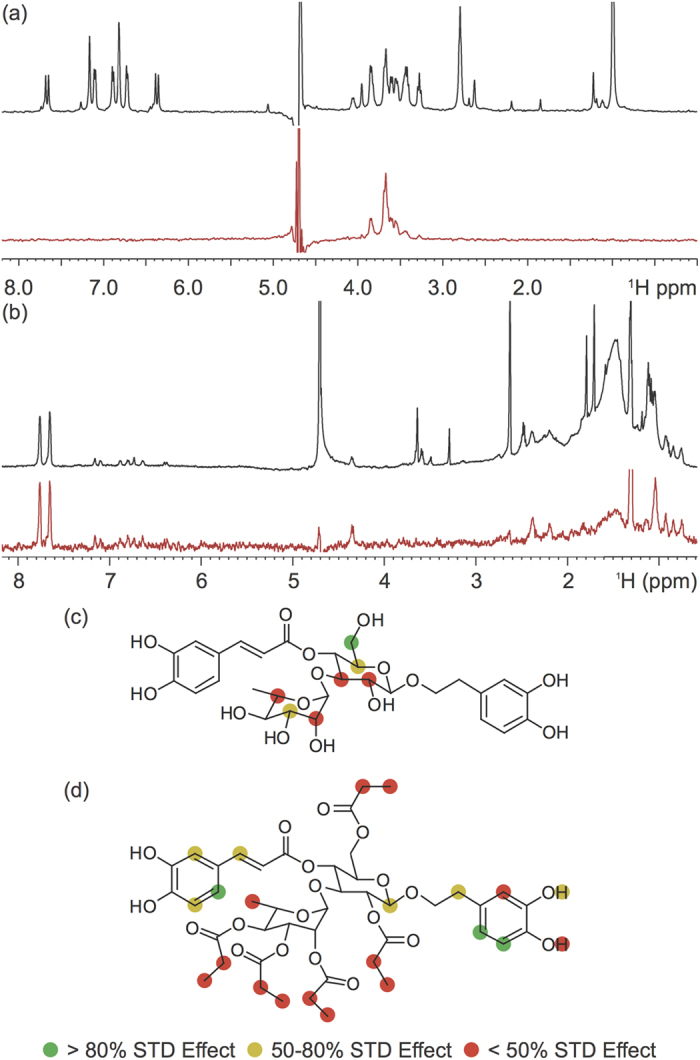
Ligand interaction with fibrillar A*β*_42_ by saturation transfer difference (STD) NMR (600 MHz). STD NMR spectra of (**a**) **Verbascoside** and (**b**) **VPP** at a 250:1 ligand to peptide ratio using pre-assembled A*β*_42_ fibrils (1 μM). Comparison of the STD signal intensity (red) to the STD reference intensity (black) reflects the relative proximity of the corresponding proton to the A*β*_42_ fibril. The STD effect calculated from relative intensities was correlated to the structure of **Verbascoside** (**c**) and **VPP** (**d**) to generate group epitope maps.

To gain further molecular level insight into the interactions between these polyphenols and different A*β* structures, saturation transfer difference (STD) NMR was employed to map the regions of both **Verbascoside** and **VPP** which bind to preformed A*β*_42_ fibrils (Fig. 6)^18^,^28^,^49^,^50^. The intensity of peaks within the STD spectra, relative to the reference spectra, is associated with the proximity of the ligand’s protons to the fibril^50^. From these values, a group epitope map can be generated, determining which protons of **Verbascoside** and **VPP** are in proximity to and binding with the fibrils. **Verbascoside** (Fig. 6c) only showed STD signals for protons linked to the glucose and rhamose rings. While most of these protons presented modest to weak signals in the STD spectrum, the protons on the C6 of the glucose ring indicated an extremely strong STD effect. For **VPP**, weak STD effect was observed around all methyl and ethyl protons of the esters appended to the sugar moieties. The stronger STD effect was observed around both the caffeic acid and hydroxytyrosol moieties, localized especially around the two aromatic protons of the catechol groups (Fig. 6d). This suggests that the esterification of the sugar rings of **Verbascoside** blocks the binding of the compound to the fibrils through the two sugar rings, redirecting their interaction to a different portion of the compound. It may be that the multiple hydroxyl groups of the sugars promote hydrogen bonds with some of the exposed side chains of the fibril; the esterification of the sugar rings could make these interactions sterically unfavorable and direct **VPP** to interact through hydrogen bonds and π–π interactions through the catechol moieties of the compound instead.

The different binding modes identified by STD experiments led us to explore the affinity of these two compounds for fibrillar A*β*. Using a fluorescence blue shift assay, the change in the fluorescence emission wavelength was monitored as fibril was titrated into solution (Fig. S6a and b). **Verbascoside** and **VPP** were shown to have an affinity of 7.80 ± 0.75 μM and 6.98 ± 0.80 μM, respectively, under the condition employed in this study. Despite very different modes of binding to the fibril, the compounds have nearly identical affinities. It should be noted that the actual affinity for the fibril in solution is likely stronger than those measured; the concentration of fibrils was calculated based on the monomer equivalent concentration. It is not possible to measure the molar concentration of fibrils in solution given their heterogeneous length and subsequently heterogeneous molecular weight. The fibril concentration is likely much lower than the monomer equivalent which would affect the concentration values used in the fit. Because the exact fibril concentration cannot be measured, however, and because the same sample of fibril was used for both titrations, we are confident that these results are internally consistent and suggest that the two compounds have very similar affinities.

The distinct difference in group epitope but similar affinity led us to investigate the binding site on the fibril for these two compounds; it was expected that two compounds with similar affinities but unique binding moieties would likely bind to separate regions of the fibril structure and therefore do not compete for binding. To probe this, we performed a competition titration experiment in which fibrils were first treated with 1 equiv of a compound (either **Verbascoside** or **VPP**) and then titrated with the other compound. The intensity of an STD peak associated with each compound (relative to the intensity of the same peak in the reference spectrum) was then used to monitor the binding of each compound to the fibril. As the competing compound is titrated into solution, if the compounds bind in the same site, it is expected that the relative intensity of the first compound’s peak will gradually decrease while the relative intensity of the titrant’s peak will increase as it competes the first compound off of the binding site. Conversely, under non-competitive binding conditions, the initial compound would maintain a constant relative intensity while the relative intensity of the titrant would increase as it binds at higher and higher concentrations. When **Verbascoside** was titrated into a solution already containing **VPP** and fibrils (Fig. S6c), it was observed that the relative intensity of the peak associated with **Verbascoside** increased during the titration while the relative intensity of the peak associated with **VPP** gradually decreased. This suggests that **Verbascoside** competes with **VPP** for a similar binding site on the fibril. The reverse of this experiment was performed (**VPP** titrated into a solution already containing **Verbascoside** and fibrils; Fig. S6d) and the same trend was observed. The relative intensity of the **VPP** peak increased while the relative intensity of the **Verbascoside** peak decreased slightly. This implies that, while the two compounds bind to the fibril using unique portions of their related structure, they are targeting a similar location on the fibril. Additionally, the blue shift assay suggests that they do so with a nearly identical affinity. This is unexpected and suggests that the esterification changes how the two compounds are able to disaggregate fibrillar species (*vide* supra) but they bind to the fibril in a relatively similar manner though using unique parts of their structure. It could be that the differing orientation of the sugar and catechol moieties of the two compounds is at least partly responsible for this difference in reactivity as STD group epitope maps reveal that the orientation is unique between compounds.

### Antioxidant Properties

**Figure 7:**
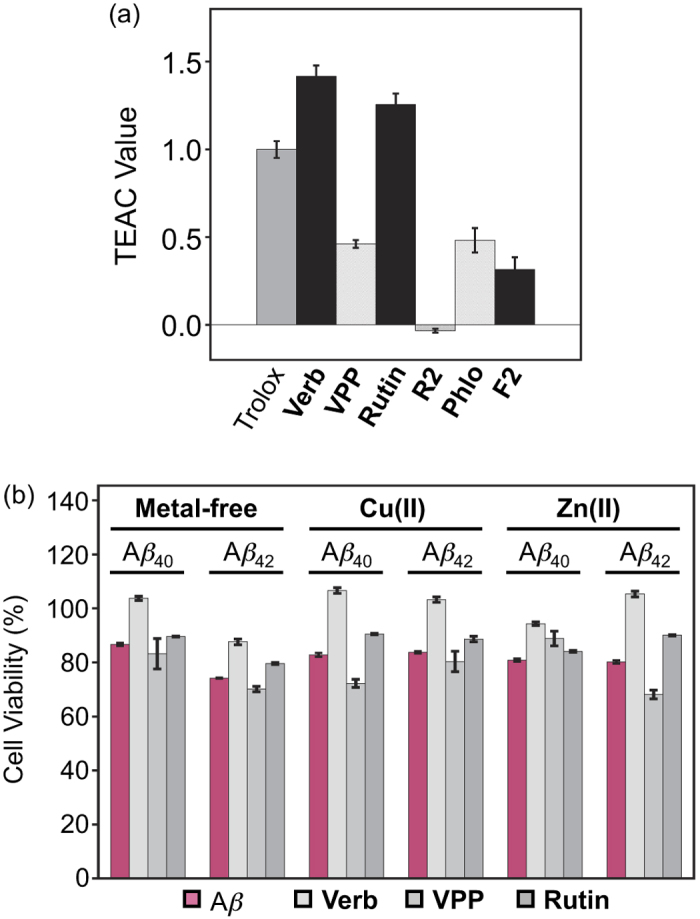
Biological activities of Verbascoside (Verb), VPP, Rutin, R2, Phlorizin (Phlo), and F2. (**a**) Free radical scavenging activity of **Verbascoside**, **VPP**, **Rutin**, **R2**, **Phlorizin**, and **F2** evaluated by a cell lysate-based TEAC assay. The TEAC values are relative to a vitamin E analogue, Trolox (6-hydroxy-2,5,7,8-tetramethylchroman-2-carboxylic acid). (**b**) Influence of **Verbascoside**, **VPP**, and **Rutin** on cytotoxicity induced by metal-free A*β* and metal-A*β* species in N2a cells. Cells treated with A*β*_40_ or A*β*_42_ (20 μM), a metal chloride salt (CuCl_2_ or ZnCl_2_; 20 μM), and a compound [**Verbascoside**, **VPP**, and **Rutin** (20 μM)] were incubated for 24 h at 37 °C. Cell viability was determined by the MTT assay. Values of cell viability (%) were calculated compared to that of cells treated with DMSO only (0–1%, v/v). Error bars represent the standard errors from three independent experiments.

Because oxidative stress is believed to play a role in AD, it would be valuable for multifunctional probes to possess antioxidant activity, on top of the ability to interact simultaneously with A*β* species and various transition metal ions^9^,^13^,^14^. The ability of these six compounds to scavenge organic radical cations (*i.e.*, ABTS^•+^) was determined by the Trolox equivalence antioxidant capacity (TEAC) assay using cell lysates (Fig. 7a)^51^,^52^. Both **Verbascoside** and **Rutin**, two known antioxidants^31^,^32^,^52^, scavenged ABTS^•+^ slightly better than Trolox (6-hydroxy-2,5,7,8-tetramethylchroman-2-carboxylic acid) (by a factor of *ca*. 1.4 and 1.2, respectively). **VPP**, **Phlorizin**, and **F2** showed a lower ability to scavenge ABTS^•+^ relative to Trolox, suggesting limited function as antioxidants relative to the other compounds investigated herein. **R2** presented no ability to scavenge ABTS^•+^. This, again, indicates that esterification of the sugar moiety transforms the function of the polyphenols. In this case, the antioxidant capacity of the all compounds was reduced upon esterification. **Verbascoside**’s activity was reduced by *ca*. 65% while **Rutin** lost all activity. The activity of **Phlorizin** was reduced by *ca*. 40%. This reduction in antioxidant capacity is surprising given the conservation of the catechol structures between **Verbascoside**/**VPP** and **Rutin**/**R2** which are thought to be potentially responsible for ABTS^•+^ quenching through semi-quinone and quinone formation^28^,^52^.

### Regulating Cytotoxicity Related to Metal-Free and Metal-Associated A*β*

Previous studies of **Verbascoside** and **Rutin** have suggested the two compounds may alleviate the toxicity of A*β* species in SH-SY5Y and APPswe cells, respectively^22^,^24^. We probed this relationship further to examine the effect of these two compounds, as well as **VPP**, on the cytotoxicity of both metal-free A*β* and metal–A*β* in murine Neuro-2a neuroblastoma (N2a) cells. In the absence of A*β*, **Verbascoside** was relatively nontoxic with and without metal ions (Fig. S7). **VPP**, however, reduced cell viability (*ca.* 70%) at high concentrations in the presence of either Cu(II) or Zn(II), but showed minimal toxicity in the absence of metal ions. **Rutin** had no significant impact on cell viability under any conditions (Fig. S6).

Cells incubated with A*β* (20 μM) in either the absence or presence of metal ions (Cu(II) or Zn(II), 20 μM) indicated viability of *ca*. 70–80% under all conditions (Fig. 7b). The addition of **Verbascoside** (20 μM) improved cell survival (*ca.* 90–100%) regardless of both A*β* isoforms and the presence or absence of metal ions. **VPP** (20 μM), however, was generally unable to regulate A*β*-triggered cytotoxicity. Finally, treatment of cells with **Rutin** (20 μM) yielded slight improvements in cell survival under all conditions. Overall, **Verbascoside** was capable of attenuating the broad toxicity indicated in the presence of both metal-free A*β* and metal–A*β*. Furthermore, esterification of the compound is shown to limit these protective capabilities.

## Discussion

Naturally occurring polyphenolic glycosides (**Phlorizin**, **Verbascoside**, and **Rutin**), along with their esterified derivatives (**F2**, **VPP**, and **R2**), were investigated for their potential to modulate the aggregation and toxicity of metal-free A*β* and metal–A*β*. Both **Verbascoside** and **VPP**, bearing multiple catechol moieties, were capable of distinctly redirecting the aggregation of metal-free A*β* (mainly, **VPP**) and/or metal–A*β* to different extents, as confirmed by both biochemical and TEM studies. The ability of these two compounds to interact with both metal ions and A*β* was confirmed through physical methods, including UV-Vis and 1D/2D/STD NMR. **Verbascoside** and **VPP** are able to interact with both Cu(II) and Zn(II) as well as with multiple forms of A*β*. The esterification of **Verbascoside** to **VPP** distinctly alters the interactions between the compounds and the components of the metal–A*β* system studied herein, suggesting that the properties (metal binding and A*β* interaction) can be tunable by synthetic modifications. In addition, **Verbascoside** is shown to have some promising chemical properties for a potential probe (antioxidant capacity, no toxicity, and cytoprotective against both metal-free A*β* and metal–A*β*). Overall, this study points to **Verbascoside** as a promising starting point for constructing a new multifunctional probe to interrogate the etiology of AD.

This study also lends insight into the efficacy of unique chemical moieties in design of anti-amyloidogenic compounds. Comparing **Phlorizin**, **Verbascoside**, and **Rutin** to each other, there is a direct correlation between the number of catechol moieties and the efficacy of each compound against metal-free A*β* and metal-A*β* aggregation. This is further supported by the group epitope map generated from STD NMR for **VPP** which shows strong STD effect around both catechols. This suggests that the catechol moiety specifically, not just polyphenols as a general class of compounds, may be effective at redirecting the protein misfolding. Previous studies have also indicated that including a catechol-like moiety makes a small molecule more effective;^28^ two of the most thoroughly investigated anti-amyloidogenic natural products, EGCG and curcumin, contain multiple variations of the catechol moiety (pyrogallol for EGCG, *o*-methyl-catechol for curcumin), further indicating that the catechol moiety is an effective component of an anti-amyloidogenic probe^20^,^53^. It should be noted, however, that catechol-containing compounds have been shown to be promiscuous in their action and functionality so their use and functionalization must be carefully considered in compound design for *in vivo* applications in order to avoid off-target effects^54^,^55^.

We have also highlighted the importance of carefully chosen synthetic alterations to efficacious reagents. In this suite of compounds, it is apparent that esterification of the sugar moieties affected the efficacy of the compounds in the various assays to differing extents; esterification increased the ability of **VPP** to alter the structure of metal-free A*β* aggregates while simultaneously maintaining its affinity for preformed fibrils relative to that of **Verbascoside**, its parent compound. Esterification also reduced the ability of all compounds to scavenge organic radicals despite not directly modifying the catechol moiety thought to be responsible for the scavenging activity. Overall, this points to the unpredictability inherent in small molecule design for complex biological systems; with many variables all contributing to the disease phenotype, it is challenging, if not impossible, to accurately predict the effects of small structural changes on a compounds efficacy against the suite of potential targets. Thus, the performance of the compounds evaluated here may serve as a benchmark for which compounds are most worthwhile investigating further in more complex and biologically relevant systems. Furthermore, it indicates the care which must be taken when functionalizing known compounds. Even potentially reversible changes, such as esterification, may drastically alter the compound’s function, in both beneficial and detrimental ways. Importantly, however, it appears from the data presented herein that **Verbascoside** possesses positive characteristics for a compound in the investigation of the role of A*β* in AD and, furthermore, is structurally amenable to a variety of future derivatization aimed at further improving its function.

## Methods

### Materials and Procedures

All reagents were purchased from commercial suppliers and used as received unless otherwise noted. The natural products (**Phlorizin**, **Verbascoside**, and **Rutin**) and their previously synthesized, esterified derivatives (**F2**, **VPP**, and **R2**) were prepared following the previously reported methods^30^,^31^,^32^. A*β*_40_ and A*β*_42_ were purchased from Anaspec (Fremont, CA, USA). Transmission electron microscopy (TEM) images were recorded on a Philips CM-100 transmission electron microscope (Microscopy and Image Analysis Laboratory, University of Michigan, Ann Arbor, MI, USA). Optical spectra for metal binding were recorded on an Agilent 8453 UV–Visible (UV–Vis) spectrophotometer. Nuclear magnetic resonance (NMR) spectra for the characterization of Zn(II) binding studies of **Verbascoside** and **VPP** were acquired on an Agilent 400 MHz NMR spectrometer. NMR studies of ^15^N-labeled A*β*_40_ with ligands were carried out on a Bruker 600 MHz NMR spectrometer equipped with a cryogenic probe. Absorbance values for biological assays, including the TEAC assay and cell viability assay, were measured on a SpectraMax M5 microplate reader (Molecular Devices, Sunnyvale, CA, USA).

### Amyloid-*β* (A*β*) Inhibition and Disaggregation Experiments

A*β* experiments were performed according to previously published methods^28^,^39^. Prior to the sample preparation, A*β*_40_ or A*β*_42_ was dissolved with ammonium hydroxide (NH_4_OH, 1% v/v, aq), aliquoted, lyophilized, and stored at −80 °C. Stock solutions (*ca*. 200 μM) of A*β*_40_ and A*β*_42_ were prepared by dissolving the lyophilized peptide in 1% NH_4_OH (10 μL) and diluting with doubly distilled (dd) H_2_O. The peptide stock solution was diluted to a final concentration of 25 μM in buffered solution containing HEPES [4-(2-hydroxyethyl)-1-piperazineethanesulfonic acid; 20 μM, pH 6.6 for Cu(II) samples; pH 7.4 for metal-free and Zn(II) samples] and NaCl (150 μM). For the inhibition studies^28^,^39^, compound (50 μM final concentration, 1% v/v DMSO) was added to the sample of A*β*_40_ or A*β*_42_ in the absence and presence of a metal chloride salt (CuCl_2_ or ZnCl_2_, 25 μM) followed by incubation for 4 and 24 h at 37 °C with constant agitation. For the disaggregation studies^28^,^39^, A*β*_40_ or A*β*_42_ with and without metal ions was first incubated for 24 h at 37 °C with continuous agitation prior to the addition of compound (50 μM). The resulting samples were incubated for an additional 4 or 24 h at 37 °C with constant agitation.

### Gel Electrophoresis with Western Blotting

The samples from the inhibition and disaggregation experiments were analyzed by gel electrophoresis with Western blot using an anti-A*β* antibody (6E10)^20^,^28^. Each sample (10 μL) was separated on a 10–20% Tris-tricine gel (Invitrogen, Grand Island, NY, USA). Following separation, the gel was transferred onto nitrocellulose membrane which was blocked with bovine serum albumin (BSA, 3% w/v, Sigma-Aldrich, St. Louis, MO, USA) in Tris-buffered saline (TBS) containing 0.1% Tween-20 (TBS-T) for 3 h at room temperature. The membrane was treated with antibody (6E10, Covance, Princeton, NJ, USA; 1:2000) in a solution of BSA (2% w/v) in TBS-T overnight at 4 °C. Following washing, the membrane was treated with horseradish peroxidase-conjugated goat antimouse secondary antibody (1:5000; Cayman Chemical, Ann Arbor, MI, USA) in 2% BSA in TBS-T solution for 1 h at room temperature. Protein bands were visualized using ThermoScientific Supersignal West Pico Chemiluminescent Substrate (Thermo Scientific, Rockford, IL, USA).

### Transmission Electron Microscopy

The samples for TEM were prepared following a previously reported method^28^,^39^. Glow-discharged grids (Formar/Carbon 300-mesh, Electron Microscopy Sciences, Hatfield, PA, USA) were treated with the samples from the disaggregation experiments (5 μL, 25 μM A*β*) for 2 min at room temperature. Excess sample was removed with filter paper and washed with ddH_2_O. Each grid was stained with uranyl acetate (1% w/v, ddH_2_O, 5 μL, 1 min), blotted to remove excess stain, and dried for 15 min at room temperature. TEM images were taken by a Philips CM-100 transmission electron microscope (80 kV, 25,000× magnification).

### Metal Binding Studies

The interaction of **Phlorizin**, **F2**, **Verbascoside**, **VPP**, **Rutin**, and **R2** with Cu(II) and Zn(II) was determined by UV-Vis or ^1^H NMR, respectively, based on previously reported procedures^20^,^56^. A solution of ligand (20 μM, pH 7.4) was prepared, treated with 0.5 to 10 equiv of CuCl_2_, and incubated at room temperature for 2 h (for **Phlorizin**, **F2**, **Verbascoside**, **VPP**, **Rutin**, and **R2**). The optical spectra of the resulting solutions were measured by UV-Vis. The interaction of both **Verbascoside** and **VPP** with ZnCl_2_ was observed by ^1^H NMR (500 MHz). ZnCl_2_ was titrated into a solution of **Verbascoside** or **VPP** (2 mM) in DMSO-*d*_*6*_ and the spectra were recorded.

### 2D NMR Spectroscopy

The interaction of A*β*_40_ with **Phlorizin**, **Verbascoside**, **VPP**, and **R2** was monitored by 2D band-Selective Optimized Flip-Angle Short Transient Heteronuclear Multiple Quantum Coherence (SOFAST-HMQC) at 8 °C^48^. Uniformly-^15^N-labeled A*β*_40_ (rPeptide, Bogart, GA, USA) was first dissolved in 1% NH_4_OH and lyophilized. The peptide was re-dissolved in 3 μL of DMSO-*d*_*6*_ (Cambridge Isotope, Tewksbury, MA, USA) and diluted with phosphate buffer, NaCl, D_2_O, and ddH_2_O to a final peptide concentration of 80 μM (20 mM PO_4_, pH 7.4, 50 mM NaCl, 7% v/v D_2_O). Each spectrum was obtained using 64 complex *t*_1_ points and a 0.1 sec recycle delay on a Bruker Avance 600 MHz spectrometer. The 2D data were processed using TOPSPIN 2.1 (from Bruker). Resonance assignment was performed with SPARKY 3.1134 using published assignments for A*β*_40_ as a guide^57^,^58^,^59^. Chemical shift perturbation (CSP) was calculated using the following equation (Eq. 1):


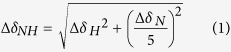


### Saturation Transfer Difference (STD) NMR Spectroscopy

For the STD NMR^48^ experiments, an 150 μM solution of fibrillar A*β*_42_ was prepared by incubating A*β*_42_ for 24 h at 37 °C with constant agitation in 10 mM deuterated Tris–DCl, 95% D_2_O at pD 7.4 (corrected for the isotope effect). The samples for STD experiments were prepared by diluting fiber to 1 μM (effective monomer concentration) into 10 mM deuterated Tris–DCl to which was added 250 μM of ligand (0.5% DMSO-*d*_*6*_). STD experiments were acquired with a train of 50 dB Gausian-shaped pulses of 0.049 sec with an interval of 0.001 sec at either −1.0 ppm (on resonance) or 40 ppm (off resonance) with a total saturation time of 2 sec on a Bruker 600 MHz NMR spectrometer. A total of 1024 scans were recorded for the STD spectrum and 512 scans were recorded for the reference spectrum at 25 °C. An inter-scan delay of 1 sec was used for both the STD and the reference experiments.

For the competition experiments with **Verbascoside** and **VPP**, the above procedure was followed for sample preparation. STD experiments were acquired with a train of 50 dB Gausian-shaped pulses of 0.049 sec with an interval of 0.001 sec at either −1.0 ppm (on resonance) or 40 ppm (off resonance) with a total saturation time of 2 sec on a Bruker 500 MHz NMR spectrometer. A total of 2048 scans were recorded for the STD spectrum and 1024 scans were recorded for the reference spectrum at 25 °C. An inter-scan delay of 1 sec was used for both the STD and the reference experiments. To a solution already containing either **Verbascoside** or **VPP** (250 μM), the other compound was titrated to 0.5 equiv (125 μM), 1 equiv (250 μM), and 3 equiv (750 μM). The intensity of peaks unique to **Verbascoside** (3.85 ppm) and **VPP** (7.78 ppm) in the STD spectra relative to their intensity in the reference spectra were used to monitor the binding of the compounds to the fiber.

### Blue Shift Fluorescence Assay

The change in the fluorescence emission wavelength of **Verbascoside** or **VPP** was monitored upon treatment with A*β*_42_ fibrils on a Fluoromax-4 Spectrofluorimeter (Horiba Scientific, Edison, NJ, USA). A 25 μM solution of either **Verbascoside** or **VPP** in buffer (20 mM PO_4_, pH 7.4, 50 mM NaCl) was titrated with preformed A*β*_42_ fibrils, prepared as described above for STD experiments. The fluorescence emission was monitored between 380 and 550 nm following excitation (350 nm for **Verbascoside** and 330 nm for **VPP**) with slits setting for 5 nm bandwidths. The blue shift was calculated by the difference between the emission maximum wavelength of the titration point and the emission maximum wavelength of the compound in absence of fibrils. The data was then fit to a hyperbolic curve to calculate the *K*_d_ value.

### Trolox Equivalent Antioxidant Capacity (TEAC) Assay

The antioxidant activity of **Phlorizin**, **F2**, **Verbascoside**, **VPP**, **Rutin**, and **R2** was determined by the TEAC assay employing cell lysate following the protocol of the antioxidant assay kit purchased from Cayman Chemical Company (Ann Arbor, MI, USA) with modifications^18^. Murine Neuro-2a (N2a) cells were used for this assay. This cell line, purchased from the American Type Culture Collection (ATCC, Manassas, VA, USA), was maintained in media containing 50% Dulbecco’s modified Eagle’s medium (DMEM) and 50% OPTI-MEM (GIBCO), supplemented with 10% fetal bovine serum (FBS, Sigma), 1% Non-essential Amino Acids (NEAA, GIBCO), 2 mM glutamine, 100 U/mL penicillin, and 100 mg/mL streptomycin (GIBCO). The cells were grown and maintained at 37 °C in a humidified atmosphere with 5% CO_2_. For the antioxidant assay using cell lysates, cells were seeded in a 6 well plate and grown to approximately 80–90% confluence. Cell lysates were prepared following the previously reported method with modifications^60^. N2a cells were washed once with cold PBS (pH 7.4, GIBCO) and harvested by gently pipetting off adherent cells with cold PBS. The cell pellet was generated by centrifugation (2,000 × g for 10 min at 4 °C). This cell pellet was sonicated on ice (5 sec pulses, 5 times with 20 sec intervals between each pulse) in 2 mL of cold Assay Buffer (5 mM potassium phosphate, pH 7.4, containing 0.9% NaCl and 0.1% glucose). The cell lysates were centrifuged at 5,000 × g for 10 min at 4 °C. The supernatant was removed and stored on ice until use. To standard and sample wells in a 96 well plate, cell lysates (10 μL) were delivered; they were followed by addition of compound, metmyoglobin, ABTS, and H_2_O_2_ in order. After 5 min incubation at room temperature on a shaker, absorbance values at 750 nm were recorded. The final concentrations (0.045, 0.090, 0.135, 0.180, 0.225, and 0.330 mM) of Trolox (Sigma-Aldrich; dissolved in DMSO) and all polyphenolic glycosides were used. The percent inhibition was calculated according to the measured absorbance [% Inhibition = (A_0_-A)/A_0_, where A_0_ is absorbance of the supernatant of cell lysates] and was plotted as a function of compound concentration. The TEAC value of ligands was calculated as a ratio of the slope of the standard curve of the compound to that of Trolox.

### Cell Viability Measurements

Cell viability upon treatment with compounds was determined using the MTT assay (Sigma). N2a cells were seeded in a 96 well plate (15,000 cells in 100 μL per well). The cells were treated with A*β* (20 μM) with or without either CuCl_2_ or ZnCl_2_ (20 μM), followed by the addition of compound (20 μM, 1% v/v final DMSO concentration for **Verbascoside**, **VPP**, and **Rutin**) and incubated for 24 h in the cells. After incubation, 25 μL MTT [5 mg/mL in phosphate buffered saline (PBS), pH 7.4, GIBCO, Grand Island, NY, USA] was added to each well and the plate was incubated for 4 h at 37 °C. Formazan produced by the cells was solubilized using an acidic solution of *N*,*N*-dimethylformamide (DMF, 50% v/v, aq) and sodium dodecyl sulfate (SDS, 20% w/v) overnight at room temperature in the dark. The absorbance was measured at 600 nm using a microplate reader. Cell viability was calculated relative to cells treated an equivalent volume of DMSO.

## Additional Information

**How to cite this article**: Korshavn, K. J. *et al.* Reactivity of Metal-Free and Metal-Associated Amyloid-β with Glycosylated Polyphenols and Their Esterified Derivatives. *Sci. Rep.* **5**, 17842; doi: 10.1038/srep17842 (2015).

## Supplementary Material

Supplementary Information
