# Supplementary material for: Reactivity of Metal-Free and Metal-Associated Amyloid-β with Glycosylated Polyphenols and Their Esterified Derivatives
Source: Sci Rep. 2015 Dec 10;5:17842. doi: 10.1038/srep17842 (PMC4674742; doi:10.1038/srep17842)
Supplement: Supplementary Information [file srep17842-s1.pdf]

Supplementary Information

for

Reactivity of Metal-Free and Metal-Associated Amyloid-β with Glycosylated Polyphenols and Their Esterified Derivatives

Kyle J. Korshavn[1], Milim Jang[2], Yeon-Ju Kwak[2], Akiko Kochi[1,2], Silvia Vertuani[3], Anirban Bhunia[4,5], Stefano Manfredini[3,*], Ayyalusamy Ramamoorthy[1,4,*] & Mi Hee Lim[2,*]

[1]Department of Chemistry, University of Michigan, Ann Arbor, Michigan 48109-1055, United States

[2]Department of Chemistry, Ulsan National Institute of Science and Technology (UNIST), Ulsan 689-798, Korea

[3]Department of Life Sciences and Biotechnology, University of Ferrara, I-44121 Ferrara, Italy

[4]Biophysics, University of Michigan, Ann Arbor, Michigan 48109-1055, United States

[5]Department of Biophysics, Bose Institute, P-1/12 CIT Scheme VII(M), Kolkata 700054, India

*To whom correspondence should be addressed: mhlim@unist.ac.kr, ramamoor@umich.edu, and mv9@unife.it

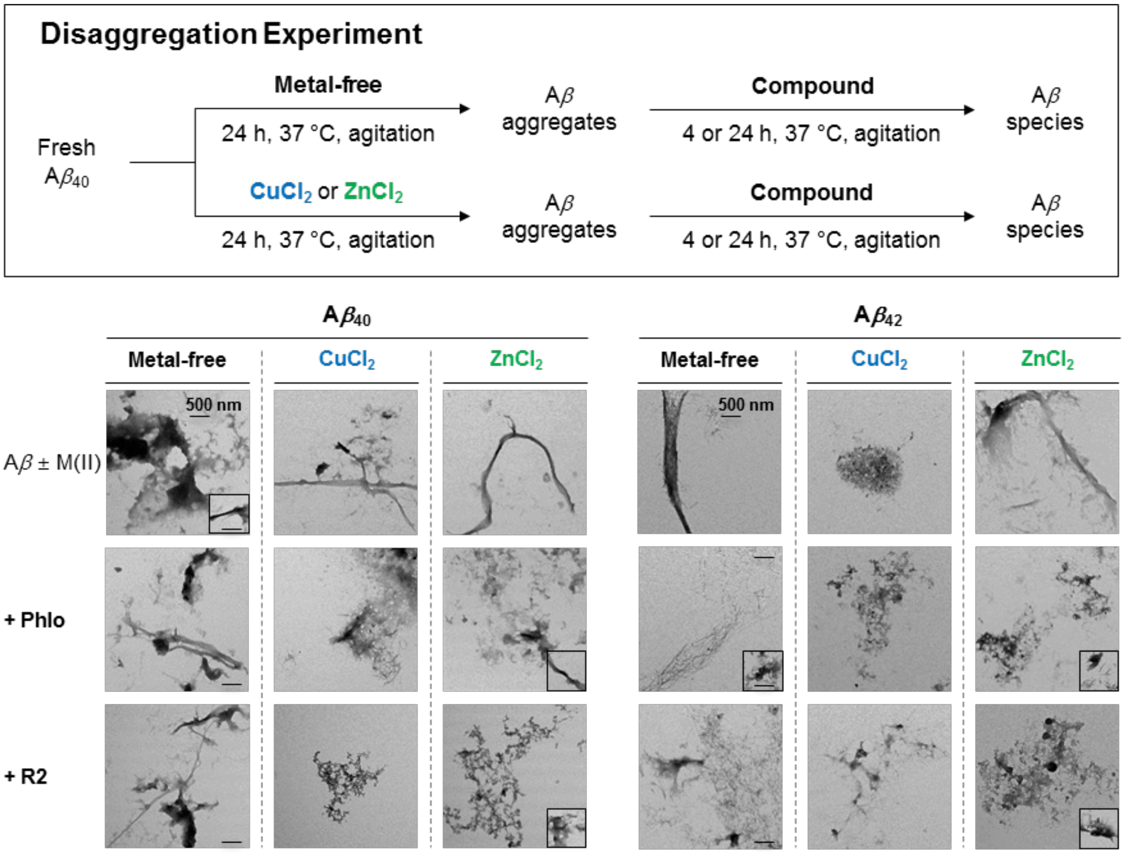

Figure S1. Visualization of the morphologies of the resultant A*β* species from the disaggregation samples that were incubated with Phlorizin or R2 for 24 h by TEM. Conditions: [Aβ] = 25 μM; [$CuCl_2$ or $ZnCl_2$] = 25 μM; [compound] = 50 μM; incubation for 4 or 24 h; pH 6.6 (for Cu(II) samples) or pH 7.4 (for metal-free and Zn(II) samples); 37 °C; constant agitation.

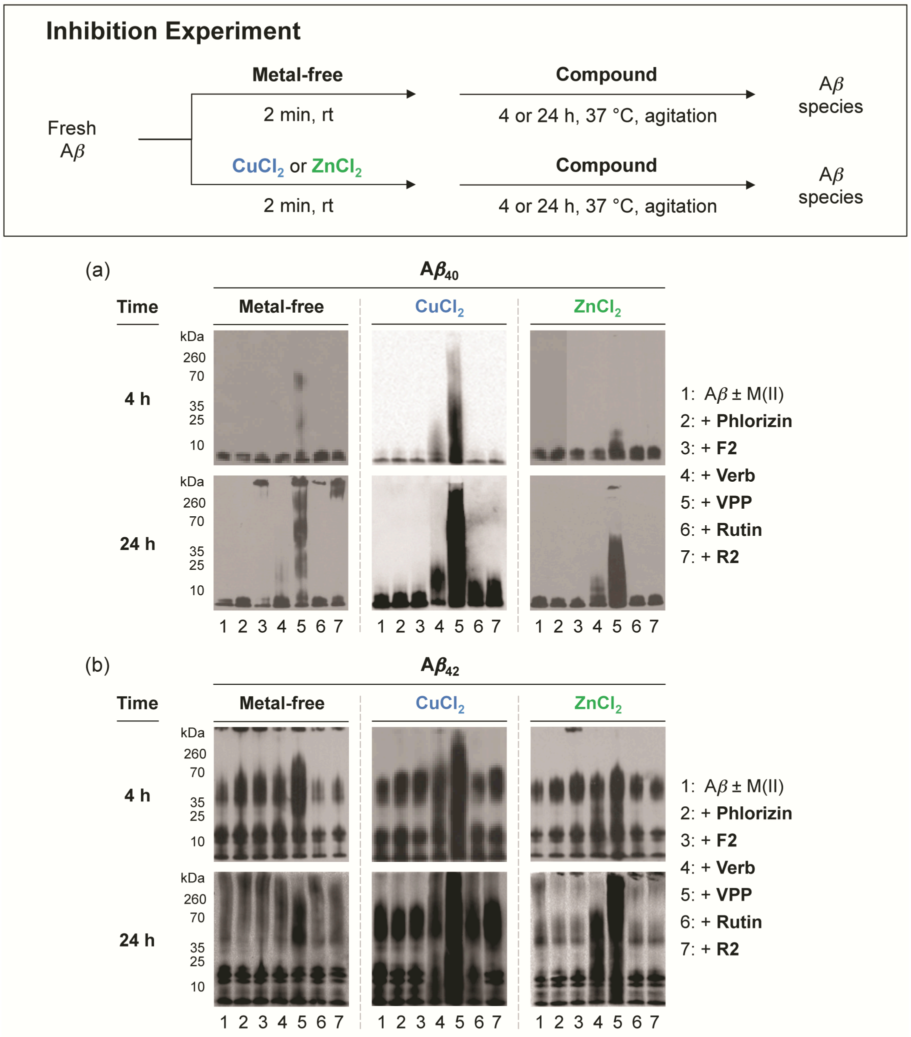

Figure S2. Effect of Phlorizin, F2, Verbascoside, VPP, Rutin, or R2 on the formation of metal-free and metal-induced A*β*$_{40}$/A*β*$_{42}$ aggregates. A*β*$_{40}$ (a) and A*β*$_{42}$ (b) were co-incubated with or without compound in both the absence and presence of either $CuCl_2$ or $ZnCl_2$ for either 4 or 24 h and visualized by gel/Western blot. Conditions: [A*β*] = 25 μM; [$CuCl_2$ or $ZnCl_2$] = 25 μM; [compound] = 50 μM; 4 or 24 h incubation; pH 6.6 (for Cu(II) samples) or pH 7.4 (for metal-free and Zn(II) samples); 37 °C; constant agitation.

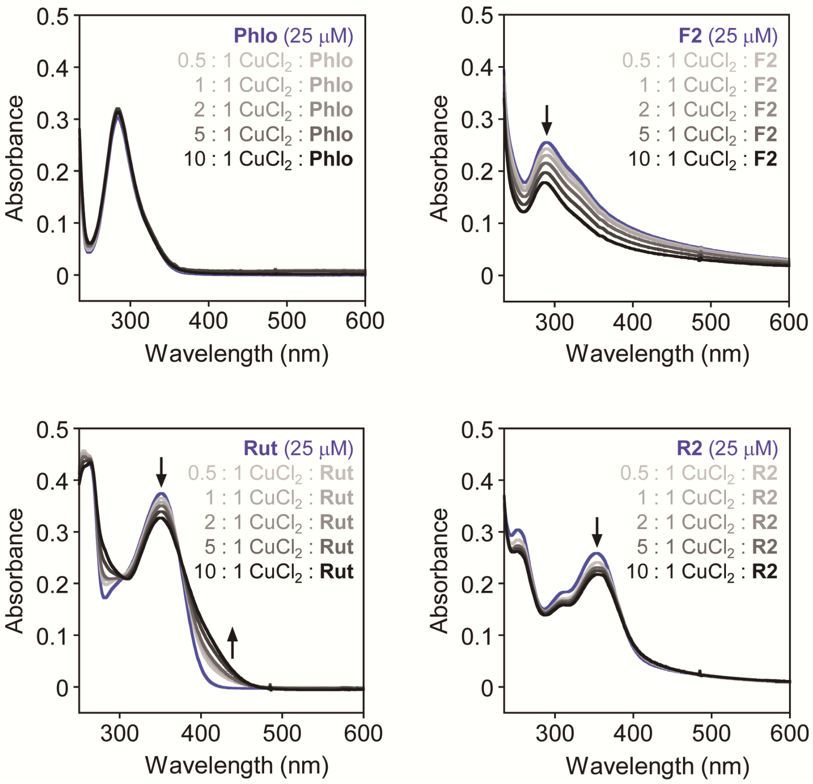

Figure S3. Cu(II) binding studies of Phlorizin (Phlo), F2, Rutin, and R2 by UV-Vis. Compounds were dissolved in buffer at pH 7.4 and titrated with Cu(II) and incubated for 2 h between reads. Experimental condition: [compound] = 25 μM; incubation for 2 h; room temperature.

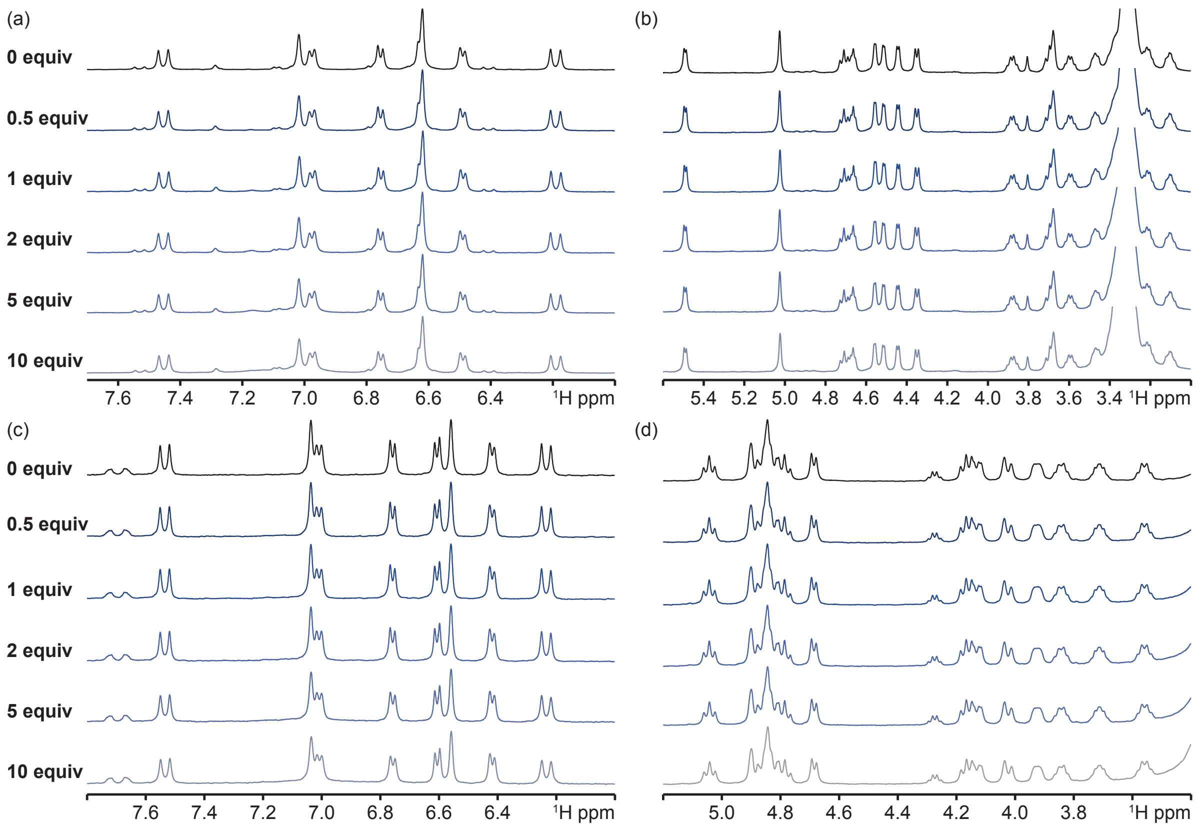

Figure S4. Zn(II) binding of non-phenolic protons of Verbascoside and VPP. Samples of (a and b) **Verbascoside** and (c and d) **VPP** were dissolved in DMSO-d_6 (2 mM) and titrated with $ZnCl_2$ (0-10 equiv). Broadening or shifts of protons in the aromatic region (a and c) and the sugar ring region (b and d) of the ^{1}H spectra were not observed over the course of the titration.

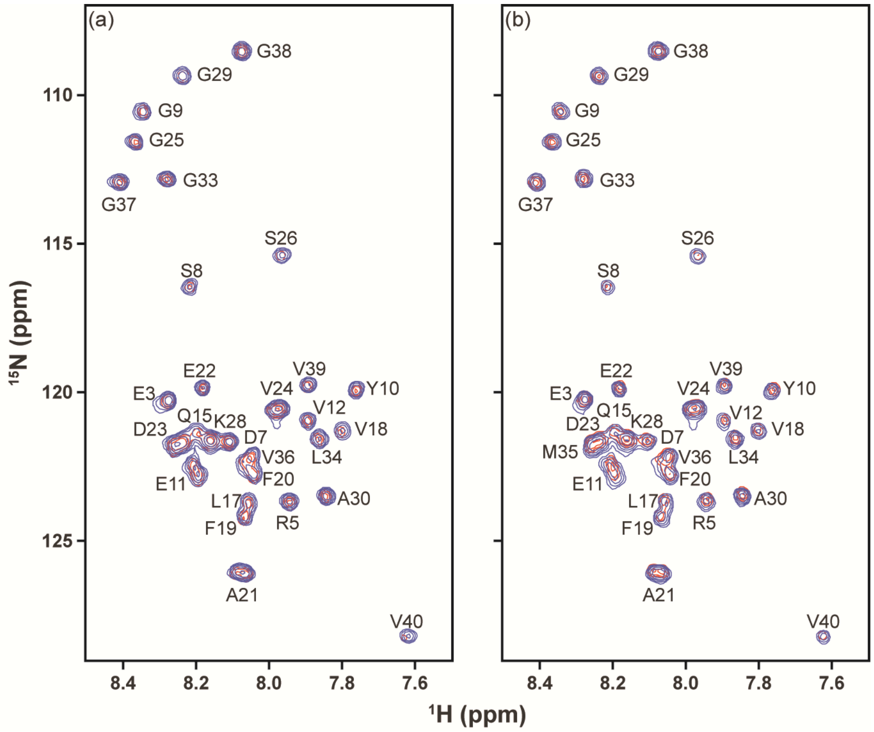

Figure S5. Interaction of Phlorizin or R2 with monomeric Aβ_{40} by SOFAST-HMQC NMR (600 MHz). (a) **Phlorizin** or (b) **R2** was titrated into a solution of uniformly ^{15}N-labeled Aβ_{40} (80 μM in 20 mM PO_4, pH 7.4, 50 mM NaCl, 7% v/v D_2O, 10 °C). The spectrum obtained upon incubation of Aβ_{40} with 10 equiv of the ligand was indicated in blue; the spectrum of Aβ_{40} only was depicted in red. All spectra were aligned at G29 due to non-uniform broadening of the water peak as a reference.

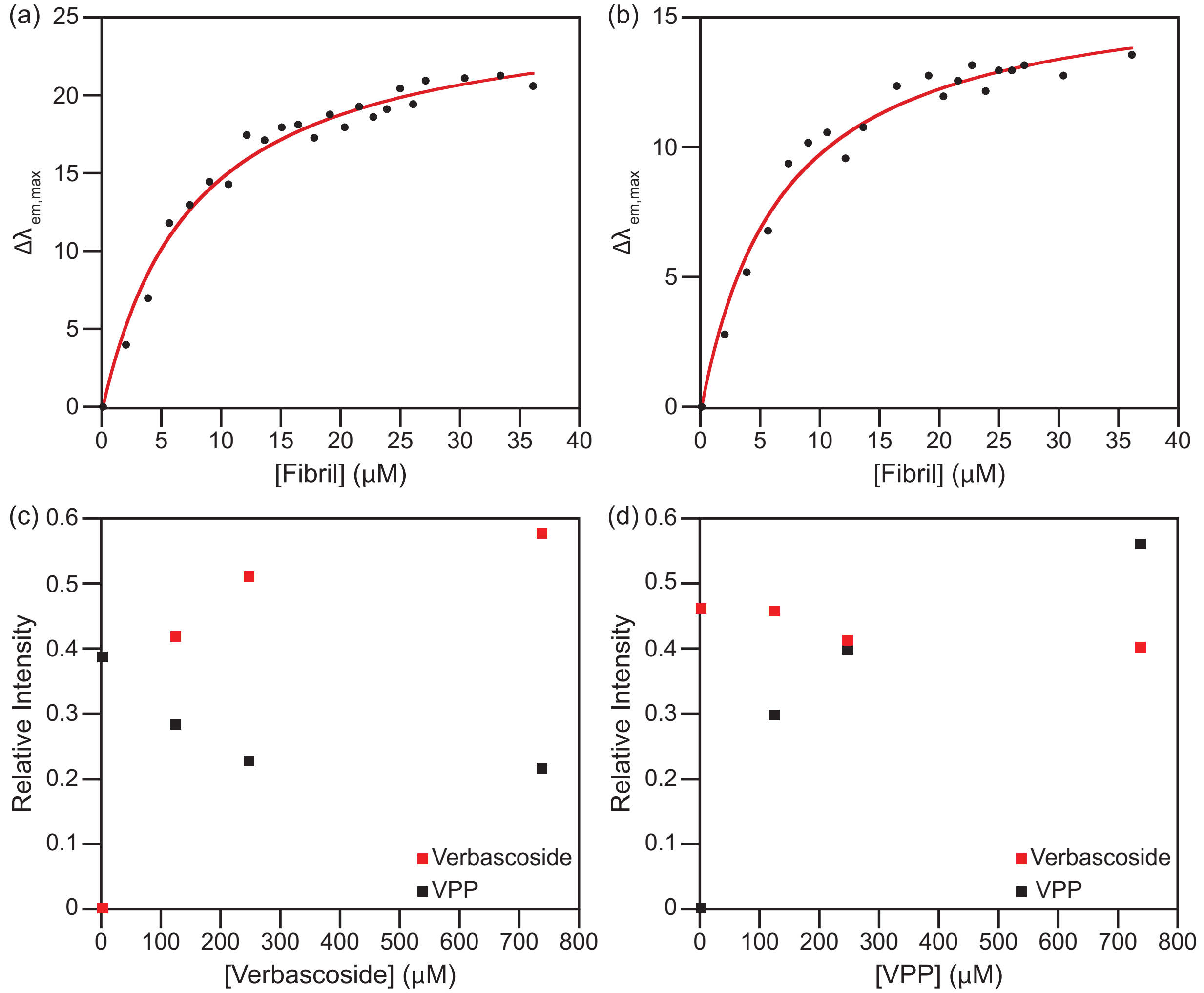

Figure S6. Affinity and competition of Verbascoside and VPP for Aβ_{42} fibrils. The affinity of (a) **Verbascoside** and (b) **VPP** for Aβ fibrils was measured by a blue shift fluorescence assay. The data were fit to a hyperbolic function to calculate the affinities (red line). The ligands were then tested for binding competition by STD. The relative intensity of the selected peaks in the STD spectra (relative to the reference spectra) were monitored over the course of a titration of (c) **Verbascoside** into the fibrils pretreated with **VPP**; (d) **VPP** into the fibrils pretreated with **Verbascoside**.

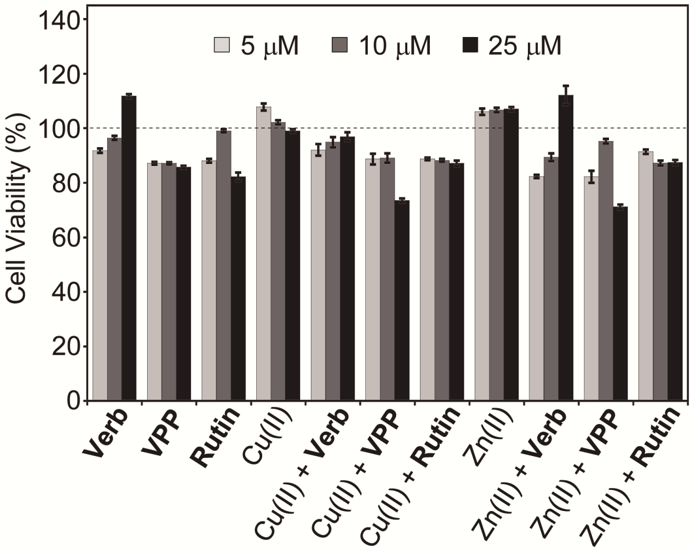

Figure S7. Cytotoxicity of Verbascoside (Verb), VPP, and Rutin in the absence and presence of $CuCl_2$ or $ZnCl_2$. N2a cells treated with a metal chloride salt ($CuCl_2$ or $ZnCl_2$; 5 μM, 10 μM, or 25 μM) and a compound [**Verbascoside**, **VPP**, or **Rutin** (5 μM, 10 μM, or 25 μM)] were incubated for 24 h at 37 °C. Cell viability was determined by the MTT assay. Values of cell viability (%) were calculated compared to that of cells treated with DMSO only (0-1%, v/v). Error bars represent the standard error from three independent experiments.
